# 0859. Altered expression of the bone morphogenetic antagonists in the bleomycin model of acute lung injury

**DOI:** 10.1186/2197-425X-2-S1-P64

**Published:** 2014-09-26

**Authors:** N Murphy, S Coyle Rowan, S Frohlich, P McLoughlin

**Affiliations:** School of Medicine and Medical Sciences, University College Dublin, Dublin, Ireland; Anaesthesia and Intensive Care Medicine, St Vincent's Hospital, Dublin, Ireland; School of Medicine and Medical Sciences, University college Dublin, Dublin, Ireland; School of Medicine and Medical Sciences, University College Dublin, Dubiln, Ireland

## Introduction

Acute lung injury (ALI) is a devastating clinical condition, characterised by acute inflammation that often proceeds to overt pulmonary fibrosis. Basal bone morphogenetic protein (BMP) signalling is essential for normal pulmonary homeostasis. The BMP antagonist gremlin has previously been shown to be increased in hypoxic lung disease, reduce BMP signalling and thus contribute directly to lung damage [1]. BMP antagonsists also play important roles in fibrotic diseases [2].

## Objectives

The aim of this study was to investigate the expresison of BMP accessory proteins in an animal model of ALI and assess any associated changes in BMP signalling.

## Methods

Acute lung injury was induced in adult mice by intra-tracheal instillation of bleomycin (1U/kg); saline was instilled in control animals. Fourteen days post inoculation, animals were anaesthetised, killed and their lungs removed and snap frozen for later analysis of RNA and protein. Lungs from separate groups were isolated, fixed, and wax embedded for histological and immunohistochemical analysis.

## Results

Bleomycin treated lungs showed characteristic structural changes and patchy inflammation. Gremlin, follistatin, follistatin like 1, follistatin like 3, BMPER and noggin mRNA expression was increased, the expression of BAMBI and NOV was reduced. MGP, KCP, lefty and chordin were unchanged. Expresion of Follistatin like 1, BAMBI and noggin protein was increased. Consistent with attentuated BMP signalling, BMP-2 and phospho-smad 1/5/8 was reduced.

## Conclusions

These data demonstrate that BMP antagonists are altered in bleomycin injured lungs and associated with reduced BMP signalling.
